# Identification of Novel Quantitative Trait Loci for Culm Thickness of Rice Derived from Strong-Culm Landrace in Japan, Omachi

**DOI:** 10.1186/s12284-023-00621-8

**Published:** 2023-01-27

**Authors:** Koki Chigira, Masanori Yamasaki, Shunsuke Adachi, Atsushi J. Nagano, Taiichiro Ookawa

**Affiliations:** 1 Graduate School of Agriculture, Tokyo University of Agriculture and Technology, 3-5-8 Saiwai-cho, Fuchu, Tokyo, 183-8509 Japan; 2 Graduate School of Science and Technology, Niigata University, 8050 Ikarashi 2-no-cho, Nishi-Ku, Niigata City, Niigata 950-2181 Japan; 3 Faculty of Agriculture, Ryukoku University, 1-5 Yokotani, Seta Oe-cho, Otsu, Shiga 520-2194 Japan; 4 Institute for Advanced Biosciences, Keio University, 403-1 Nipponkoku, Daihouji, Tsuruoka, Yamagata 997-0017 Japan

**Keywords:** Lodging resistance, Temperate *japonica* rice, Landrace, QTL, Genome breeding

## Abstract

Increasing the lodging resistance of rice through genetic improvement has been an important target in breeding. To further enhance the lodging resistance of high-yielding rice varieties amidst climate change, it is necessary to not only shorten culms but strengthen them as well. A landrace rice variety, Omachi, which was established more than 100 years ago, has the largest culm diameter and bending moment at breaking in the basal internodes among 135 temperate *japonica* accessions. Using unused alleles in such a landrace is an effective way to strengthen the culm. In this study, we performed quantitative trait locus (QTL) analysis to identify the genetic factors of culm strength of Omachi using recombinant inbred lines (RILs) derived from a cross between Omachi and Koshihikari, a standard variety in Japan. We identified three QTLs for the culm diameter of the 5th internode on chromosomes 3 (*qCD3*) and 7 (*qCD7-1*, *qCD7-2*). Among them, *qCD7-2* was verified by QTL analysis using the F_2_ population derived from a cross between one of the RILs and Koshihikari. RNA-seq analysis of shoot apex raised 10 candidate genes underlying the region of *qCD7-2*. The increase in culm strength by accumulating Omachi alleles of *qCD3*, *qCD7-1* and *qCD7-2* was 25.0% in 2020. These QTLs for culm diameter pleiotropically increased spikelet number per panicle but did not affect days to heading or culm length. These results suggest that the Omachi alleles of *qCD3*, *qCD7-1* and *qCD7-2* are useful for breeding to increase lodging resistance and yield.

**Supplementary Information:**

The online version contains supplementary material available at 10.1186/s12284-023-00621-8.

## Background

Rice is a staple food consumed by more than half of the world population. Asia in particular accounts for about 90% of the world’s rice production (Muthayya et al. 2014; Bandumula 2018). Rice-producing regions in Asia are susceptible to damage from typhoons and rainstorms, causing lodging and severely affecting rice production (Ishimaru et al. 2008; Lou et al. 2012; Blanc and Strobl 2016), so lodging resistance has been an important target of breeding for modern rice varieties. In landrace rice, the culm elongates substantially under high fertilization conditions. These varieties are prone to lodging under modern production methods. In the Green Revolution of the 1960s, varieties with the semi-dwarf allele, *sd1*, was introduced to prevent lodging even under increased fertilizer application (Ashikari et al. 2002; Sasaki et al. 2002). This semi-dwarfism resulted in a large increase in grain yield with the improvement in plant architecture (Wang et al. 2020). However, because shortening plant height restricts yield potential, breeding cannot rely upon semi-dwarfism alone to further yield (Zhu et al. 2016). In high-yielding varieties especially, the introduction of genes increasing number or size of grain has resulted in heavier panicles, but the culms supporting those panicles have not been improved, and there is a concern that lodging will become a problem again (Hirano et al. 2017). Furthermore, severe typhoons and heavy rainfall in the East Asia are expected to increase in the future (Mishra 2019; Ishii and Mori 2020; Sugi et al. 2020; Yamaguchi et al. 2020). To cope with this situation, enhancing the mechanical strength of the culm is a crucial target for improving lodging resistance.

The mechanical strength of culms is quantified by bending moment at breaking (BM). BM is composed of bending stress (BS), which is an index of culm stiffness, and section modulus (SM), which is an index of culm thickness (Ookawa and Ishihara 1992). BS is associated with properties such as the morphology of cortical fibre tissue and the cell wall components such as lignin, cellulose and hemicellulose (Matsuda et al. 1983). SM is calculated from the inner and outer diameters of culm.

Identifying quantitative trait loci (QTLs) for these traits from a set of genetic resources is a promising approach for improving culm mechanical strength through genome breeding. From the chromosome segment substituted lines (CSSLs) derived from *indica* variety, Habataki, and temperate *japonica* variety, Sasanishiki, *STRONG CULM 1 (SCM1)* and *SCM2* have been identified as QTLs of bending moment at breaking (BM) and section modulus (SM) (Ookawa et al. 2010). The causal gene for *SCM2* is *Aberrant Panicle Organization 1 (APO1)*, which encodes an F-box-containing protein involved in morphogenesis of the panicle. The Habataki allele of *APO1* enhances culm strength as well as grain yield. From the back-cross inbred lines derived from the tropical *japonica* variety, Chugoku 117, and temperate *japonica* variety, Koshihikari, *SCM3* and *SCM4* have been identified as QTLs of BM and SM, and the causal gene for *SCM3* is *Fine Culm 1 (FC1*), which encodes a transcription factor involved in strigolactone signaling (Yano et al. 2015a). The Chugoku 117 allele of *FC1* thickens culms and increases the number of grains per panicle, but decreases the number of panicles and has no effect on yield. In addition, a QTL for pushing resistance of the lower part of the plant, *prl5*, was identified from the *indica* cultivar, Kasalath (Kashiwagi and Ishimaru 2004). *BSUC11*, the QTL related to bending-type lodging of upper part of culms, was also identified from Kasalath (Kashiwagi 2014). Most existing studies used a set of genetic populations derived from distantly related varieties such as *indica* and *japonica*, and tropical *japonica* and temperate *japonica*. However, few genes that explain the diversity of culm strength within temperate *japonica* subspecies have been identified. QTLs related to culm strength have additive effects, and accumulating multiple superior alleles is necessary for improving culm strength sufficiently (Ookawa et al. 2022). Therefore, identifying unused alleles present within the temperate *japonica* subspecies is a promising strategy to further improve culm strength.

Landrace rice varieties potentially have useful alleles for improving culm strength of the modern varieties. In our previous studies, we evaluated the culm strength and morphological traits of 135 temperate *japonica* rice varieties, including landraces and modern varieties, and found that the culms of landraces were consistently thicker than those of modern varieties (Chigira et al. 2020). This tendency has also been observed in research on other populations (Nomura et al. 2021), which suggests that the landraces have culm-strength enhancing alleles that modern varieties do not have. However, the subsequent genome-wide association study (GWAS) for culm thickness has yet to clarify the genetic effects of the alleles that originate from the landrace, where the phenotypic variance explained (PVE) of the QTL was 7.6 to 24.5% (Chigira et al. 2020). In the field of human genetics, where GWAS has been used from early on, there exists a problem called ‘missing heritability,’ in which phenotype-associated variations obtained by GWAS cannot explain the actual heritability (Manolio et al. 2009). One of the reasons for this phenomenon is thought to be that quantitative traits are affected by many rare mutations (Young 2019).

In terms of the availability of alleles, identifying alleles that have not been used in past breeding is crucial for future breeding. Another requirement for alleles for improving culm strength is that they do not affect culm length. An example of a gene affecting culm strength and culm length is *SD1*. The functional *SD1* allele enhances culm thickness compared to the semi-dwarf allele, *sd1* (Ookawa et al. 2016). However, the *SD1* allele also increases culm length, so it is difficult to use in breeding to improve lodging resistance.

To identify such useful alleles, we focused on Omachi, which had the strongest culm in our previous GWAS panel of 135 varieties. We analyzed its characteristics associated with a strong culm and identified the QTLs specific to Omachi using a bi-parental population. Omachi is one of the landrace rice varieties in Japan and continues to be cultivated as a premium variety for brewing Japanese rice wine (*sake*) due to its large grain size and white core in the grain (Arai-Kichise et al. 2011). Due to its tall plant height and long panicle, it is susceptible to bending-type lodging but it has resistance to breaking-type lodging due to its thickness. Omachi is sometimes used for breeding *sake* rice varieties, but it is rarely used as a breeding material for modern varieties for staple food. This means that many modern varieties, including those not only for *sake* brewing but also for food or feed, could be improved by introducing new alleles that confer the culm strength of Omachi.

In this study, we report the characteristics of Omachi and its QTLs that are useful for breeding to enhance culm strength. Because traits related to culm strength tend to be pleiotropically expressed in other traits related to yield potential, we analyze for culm strength, as well as for yield component traits and other morphological traits. In addition, we explore QTLs related to weight and shape of grain associated with *sake* brewing quality that give the unique characteristics of Omachi and examine their relationship with culm strength.

## Results

### Characterization of Properties for Culm Strength of Omachi

**Fig. 1:**
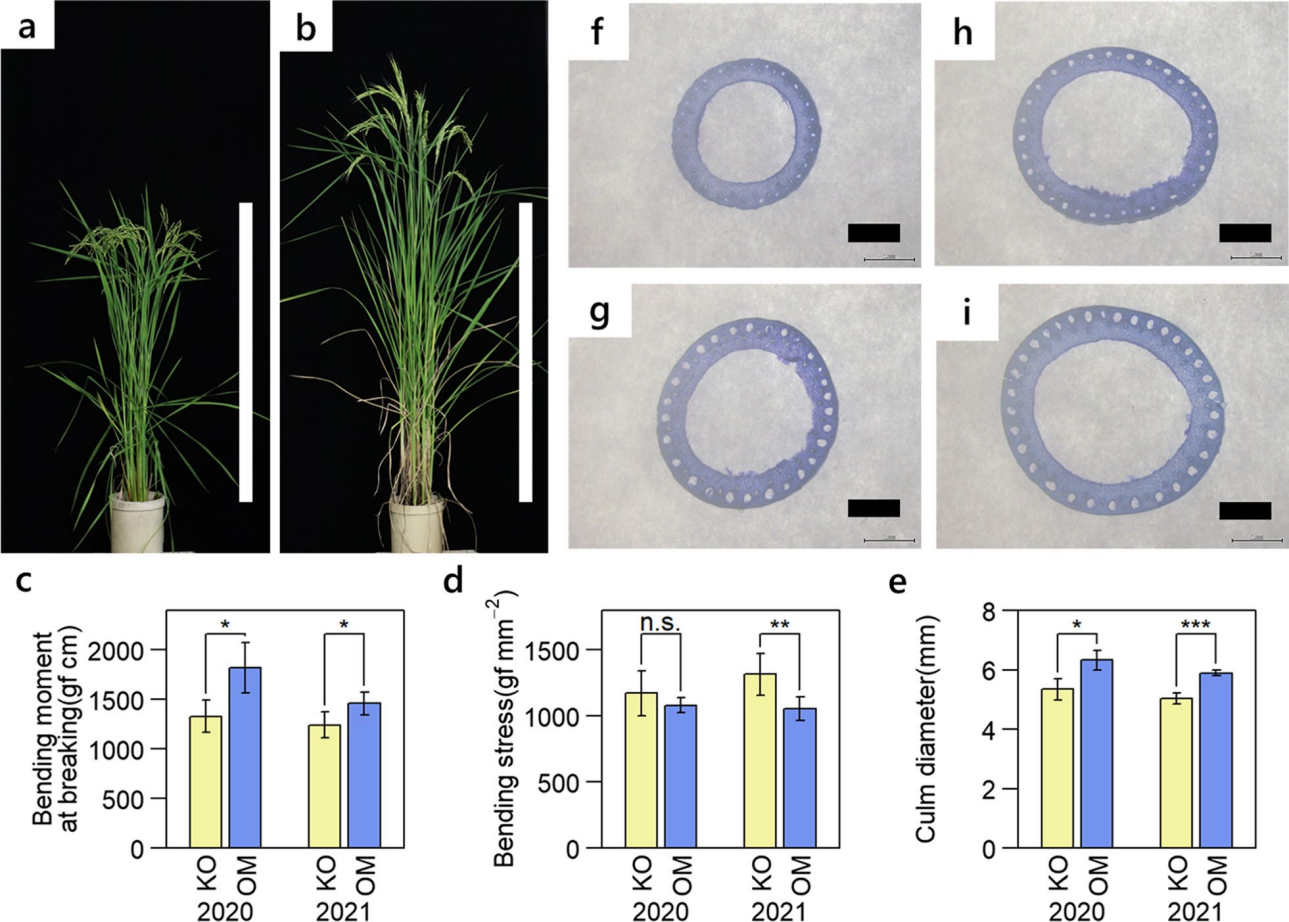
Comparison of traits associated with culm strength in Koshihikari and Omachi. **a**, **b** Plant appearances of (**a**) Koshihikari and (**b**) Omachi. White bar indicates 1 m. **c**–**e**. Comparison of the traits related to culm strength in the 5th internode. The values indicate averages of 3 and 6 replications in 2020 and 2021, respectively. Error bars indicate standard deviation. A t-test was performed assuming equal variances between two varieties, and *p* < 0.001, *p* < 0.01, *p* < 0.05, *p* ≥ 0.05 were marked with ***, **, *, n.s., respectively. **f**–**i**. Picture of cross-sections in (**f**) the 4th internode of Koshihikari, **g** the 5th internode of Koshihikari, **h** the 4th internode of Omachi, and **i** the 5th internode of Omachi. Black bar indicates 2 mm.

We first compared morphological properties between Omachi and Koshihikari. The culm length (CL) of Omachi was 18% (2020) and 26% (2021) longer than that of Koshihikari, and the panicle length (PL) were 5.9 cm (2020) and 2.8 cm (2021) longer than those of Koshihikari (Fig. 1a and b, Additional file 2: Table S1). The awn length (AL), flag leaf length (FL), number of secondary branches (SBN), and number of spikelets per panicle (SN) of Omachi were also significantly larger than that of Koshihikari in both years (Additional file 2: Table S1). In contrast, the number of panicles (PN) in Omachi was significantly lower than that of Koshihikari in both years (Additional file 2: Table S1).

Bending moment at breaking (BM), which is a mechanical index of culm strength, is the product of bending stress (BS), which is an index of culm stiffness, and section modulus (SM), which is an index of culm thickness (Ookawa and Ishihara 1992). SM is calculated from the inner and outer diameters of culm, and there are strong correlations between the SM and the inner and outer diameters in temperate *japonica* varieties (Chigira et al. 2020). Therefore, we used culm diameter (CD) as the trait indicating culm thickness in this study. The BM of Omachi was significantly larger than that of Koshihikari by 37% (2020) and 17% (2021) (Fig. 1c, Additional file 2: Table S1). The BS of Omachi was similar to that of Koshihikari in 2020 and lower in 2021 (Fig. 1d). The CD of the 5th internode (CD5) of Omachi was significantly larger than that of Koshihikari by 17% (2020) and 18% (2021) (Fig. 1e–i, Additional file 2: Table S1). These results indicate that the higher culm strength of Omachi is attributed to its thicker culm.

The differences of grain size, weight and white core trait were investigated, because these traits closely relate to the suitability for *sake* brewing of Omachi (Additional file 2: Table S1). The thousand grain weight (TGW) was significantly higher in Omachi by 25% (2020) and 15% (2021) (Additional file 2: Table S1). The grain width (GW) and the grain length (GL) were also larger in Omachi (Additional file 2: Table S1). There was no significant difference in the white core rate, despite the grains of Omachi are generally considered to have a white core (Yoshida 2012).

The weather conditions during the experiment were different in both years. In 2021, low temperatures and little sunshine had been continued from August 12 to 18 and September 1 to 9 (Additional file 1: Fig. S1).

### Genetic Variation in Recombinant Inbred Lines (RILs)

**Fig. 2:**
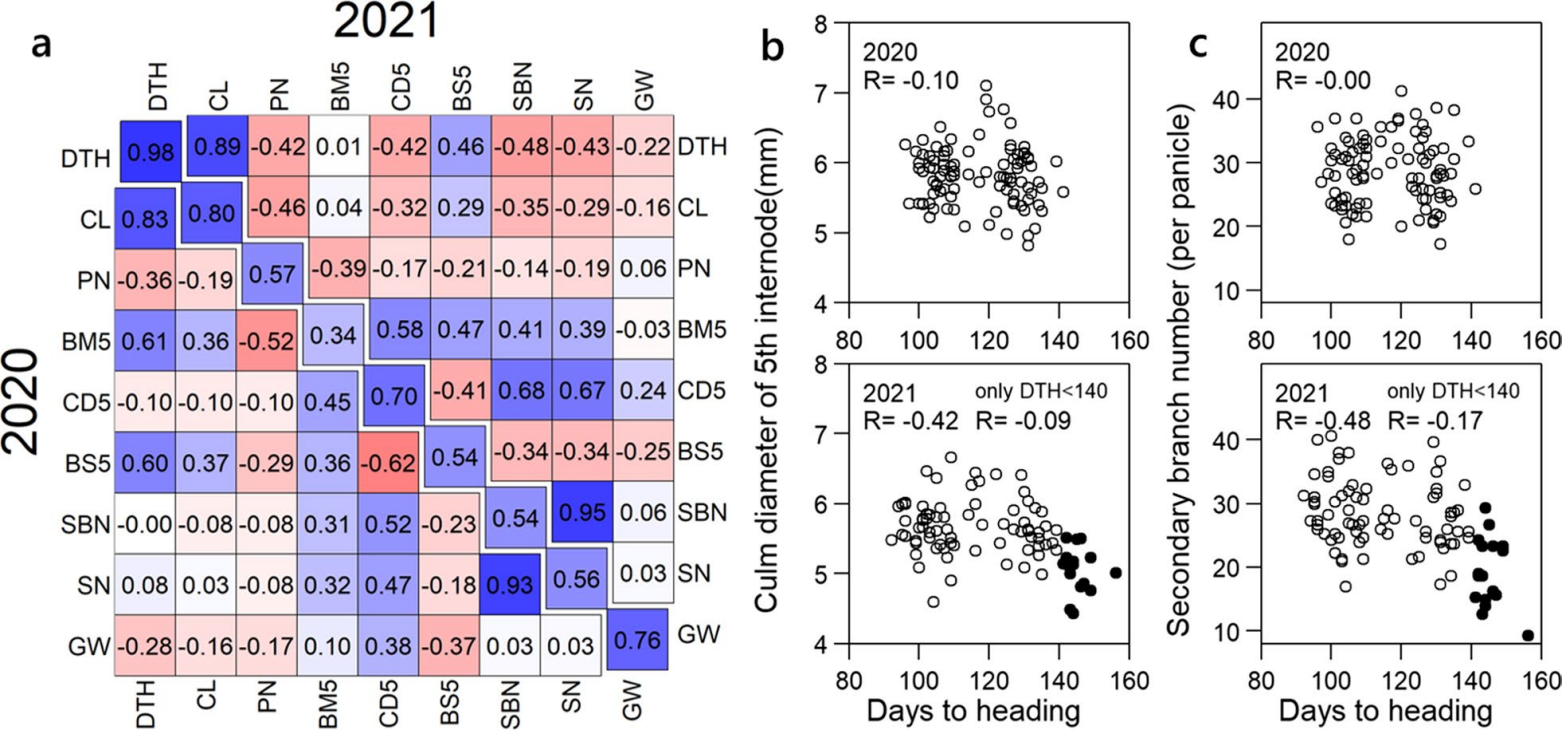
Correlation among the traits in RILs. **a** Correlation matrix among extracted traits in RILs. The lower and upper triangular matrix shows the correlation coefficient in 2020 and 2021, respectively. Numbers on the diagonal indicate correlation coefficients between two years. A figure with all traits is shown in Additional file 1: Fig. S3. **b**, **c** Correlation between DTH and CD5 (**b**), and between DTH and SBN (**c**). Top: 2020, bottom: 2021. Black plots indicate lines which omitted from QTL analysis (lines whose DTH were over 140). Abbreviations: DTH: Days to heading, CL: Culm length, PN: Number of panicles, BM5: Bending moment at breaking of 5th internode, CD5: Culm diameter of 5th internode, BS5: Bending stress of 5th internode, SBN: Number of secondary branches, SN: Number of spikelets per panicle, GW: Grain width

We developed a set of RILs population (F_14_ in 2020, F_15_ in 2021, *n* = 96) derived from the cross of Koshihikari × Omachi. Each trait showed a wide distribution among the RILs (Additional file 2: Table S2). For days to heading (DTH), the distribution was divided into two peaks (Additional file 1: Fig. S2). The same trend was also observed in CL, which are highly correlated with DTH (Fig. 2, Additional file 1: S2). The other traits generally showed a normal distribution, suggesting that multiple QTLs control these traits (Fig. 2, Additional file 1: S2).

We examined the correlations between the two experimental years to determine the stability of the traits (Fig. 2a, Additional file 1: S3). The correlation coefficient between the two years was 0.98 for DTH, followed by 0.80 for CL (Fig. 2a). CD5 also showed a strong positive correlation (R = 0.70) between both years (Fig. 2a). In contrast, there was a low correlation coefficient between the two years for BM (R = 0.34) (Fig. 2a). Among the traits related to culm strength, CD5 was stably expressed, and is expected to have a high detection rate of QTLs.

We examined the correlations between pairs of traits. Traits related to axial growth, such as CL, AL, and FL, had strongly positive correlations with DTH (Fig. 2a, Additional file 1: S3). In contrast, traits related to radial growth, for example, CD5 and SBN, had little correlation with DTH in 2020 (− 0.10 and − 0.00, respectively) (Fig. 2b, c). In 2021, they had significant negative correlations. However, when 17 lines with DTH exceeding 140 days were excluded, the correlation coefficients were − 0.09 and − 0.17 for CD5 and SBN, respectively (Fig. 2b, c). This indicates that the late maturity lines had a negative effect on CD5 and SBN in 2021. As mentioned above, there were two unseasonable weather events in 2021, and these periods corresponded to the panicle formation and internode elongation stages for lines with DTH > 140. Therefore, the traits associated to culms and panicles of these lines were considered to have been influenced by unseasonable weather. We also excluded them from subsequent QTL analysis to prevent false detection of QTLs.

The correlation coefficients between CD5 and SN were 0.47 in 2020, and 0.67 in 2021, although those between CD5 and PN were − 0.10 in 2020, and − 0.17, respectively (Fig. 2a). This suggests that the genetic factors determining CD5 may pleiotropically increase SN, while the negative impact on PN is minor.

There was no positive correlation between CD5 and CL over two years, suggesting that these two traits are controlled by different genes (Fig. 2a).

### QTL Analysis

We performed QTL analysis using all phenotypic data collected in both years. As a genotype data, 1,904 DNA markers obtained from a GoldenGate® Assay (Illumina, CA, USA) and GRAS-Di® analysis (TOYOTA, Aichi, Japan) were used (Additional file 1: Fig. S4). We excluded the 17 lines with more than 140 DTH from the phenotype data in 2021 because these lines had a large effect on traits due to the delay in heading as mentioned in the example of CD5.

In total, 27 significant QTLs were identified across 16 traits, and among them, 14 QTLs were detected in both years (Table 1). The QTLs for DTH were detected in the long arm of chromosome 3 and the short arm of chromosome 6 (Table 1). The former (*qDTH3*) contained *Hd6* at 31.51 Mb and *Hd16* at 33.00 Mb, and the latter (*qDTH6*) contained *Hd17* at 2.24 Mb (Takahashi et al. 2001; Matsubara et al. 2012; Hori et al. 2013). The alleles of these three genes were different between Koshihikari and Omachi (Additional file 1: Fig. S5). This strongly suggests that the difference in DTH between Koshihikari and Omachi is controlled by these three genes. QTLs were also detected at the same or close to the same position of *qDTH3* as CL, PL, AL, FL, PN, BM, BS, and length of the 4th internode. Considering the strong correlation between these traits and DTH, the genes for flowering in *qDTH3* should have pleiotropic effects on these multiple traits. In the internode length (IL) and the number of primary branches (PBN), QTLs were detected at different locations than *qDTH3* (Table 1). However, they were inconsistent over the two years.

**Table 1:** List of significant QTLs for all traits

Trait	Chr	QTL name	Inter-node	2020				2021				Known gene
				LOD	Physical position(Mbp)	PVE (%)	Additive effect^a^	LOD	Physical position (Mbp)	PVE (%)	Additive effect	
DTH	3	*qDTH3*		28.16	29.09	71.6	11.14 ± 0.62	25.77	29.09	64.8	15.93 ± 1.00	*Hd6*, *Hd16*
	6	*qDTH6*		4.29	0–3.06	5.0	− 2.77 ± 0.59	3.99	0–3.47	5.1	− 4.34 ± 0.97	*Hd17*
CL	3	*qCL3*		14.89	29.09–34.61	51.1	5.85 ± 0.59	9.13	29.09–34.61	43.7	6.43 ± 0.83	*Hd6*, *Hd16*
PL	3	*qPL3*		8.33	29.09–34.61	32.9	0.92 ± 0.14					*Hd6*, *Hd16*
AL	3	*qAL3*		8.18	29.09–34.61	32.5	0.73 ± 0.11	4.13	32.35–36.35	23.3	0.72 ± 0.15	*Hd6*, *Hd16*
FL	3	*qFL3*		8.68	29.09–34.61	34.1	3.63 ± 0.52	7.99	29.09–34.61	39.1	4.86 ± 0.69	*Hd6*, *Hd16*
PN	3	*qPN3*		3.49	29.09–34.61	13.4	− 0.47 ± 0.12	4.26	29.09	16.4	− 0.81 ± 0.18	*Hd6*, *Hd16*
	7	*qPN7*						4.69	24.57–26.64	15.1	− 0.71 ± 0.16	
BM	3	*qBM3*	IV	13.04	29.09–34.61	46.5	157.6 ± 17.4	5.90	29.09–34.61	29.1	103.6 ± 18.4	*Hd6*, *Hd16*
			V	5.75	29.09–34.61	27.9	136.5 ± 24.7	7.19	29.09–34.61	46.5	259.0 ± 38.9	
BS	3	*qBS3*	IV	11.81	29.09–36.35	43.3	211.7 ± 25.0	5.90	29.09–34.61	29.1	142.9 ± 25.4	*Hd6*, *Hd16*
			V	3.58	35.13	18.4	97.2 ± 23.0	3.46	35.13	26.0	151.4 ± 35.8	
CD	3	*qCD3*	V					4.94	21.64–24.26	26.2	0.22 ± 0.04	
	7	*qCD7-1*	IV					3.43	1.78–2.36	18.1	0.15 ± 0.04	
	7	*qCD7-2*	IV	3.67	24.57–26.64	16.1	0.14 ± 0.03					
			V	3.96	23.82–24.70	17.3	0.18 ± 0.04	3.62	21.94–26.64	15.0	0.16 ± 0.04	
IL	3	*qIL3*	IV					4.22	27.42–28.65	21.8	0.73 ± 0.16	*Hd6*, *Hd16*
			V	16.38	29.09–34.61	61.3	2.41 ± 0.19	6.63	29.09 –34.61	32.1	1.65 ± 0.27	
	7	*qIL7*	V	3.44	23.69–28.17	8.8	− 0.93 ± 0.20					
	12	*qIL12*	IV	3.52	19.47–21.48	15.5	− 0.66 ± 0.16					
PBN	2	*qPBN2*		3.96	20.98–24.52	14.5	− 0.36 ± 0.08					
	3	*qPBN3*		3.36	6.74–8.23	12.4	0.34 ± 0.08					
	5	*qPBN5*						3.41	27.73–28.90	18.0	0.65 ± 0.16	
SBN	7	*qSBN7*		6.36	24.57–26.64	26.3	2.72 ± 0.47					
SN	7	*qSN7*		6.00	24.57–26.64	25.0	10.32 ± 1.84	4.17	24.57–26.64	21.6	10.81 ± 2.35	
TGW	10	*qTGW10*						3.40	20.91	15.2	− 0.60 ± 0.14	
	11	*qTGW11*						4.26	16.96–20.16	30.8	0.85 ± 0.14	
GL	3	*qGL3*						5.50	29.09–34.61	18.5	− 0.12 ± 0.02	*Hd6, Hd16*
	11	*qGL11*		7.03	17.41–20.16	29.4	0.10 ± 0.02	4.66	16.96–24.25	19.9	0.14 ± 0.03	
GW	5	*qGW5*		5.33	27.73–28.90	16.0	0.04 ± 0.01	3.90	27.73–28.90	16.6	0.04 ± 0.01	
	6	*qGW6*		3.22	26.12–30.97	11.3	0.03 ± 0.01	3.90	25.65–30.97	15.3	0.04 ± 0.01	

^a^Additive effect: Positive value indicates that trait value is higher in the RILs with Omachi allele than in those with Koshihikari allele

**Fig. 3:**
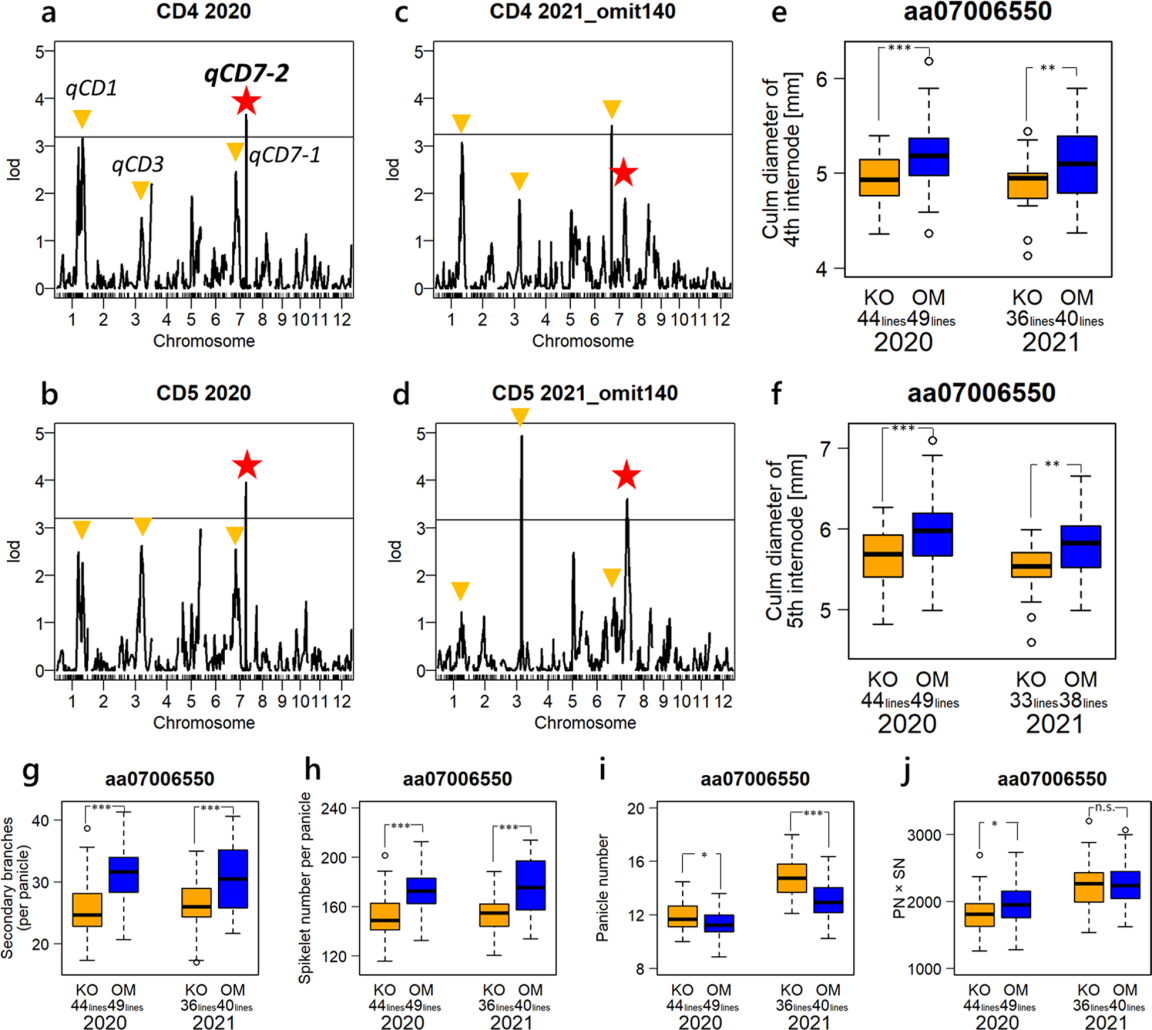
QTL analysis of culm diameter and trait values by alleles in *qCD7-2*. **a**, **d**: Result of QTL analysis of culm diameter: **a** 4th internode in 2020, **b** 5th internode in 2020, **c** 4th internode in 2021, **d** 5th internode in 2021. All 96 RILs were used for the QTL analysis in 2020 but the QTL analysis in 2021 was performed on 79 lines, excluding 17 lines with DTH exceeding 140 days. A peak marked with a red star is a major QTL, *qCD7-2*. Other peaks marked with orange triangles are suggestive QTLs. **e**–**j** Trait values by alleles in *qCD7-2* It is classified by genotype in “aa07006550”, which is a representative SNP marker for *qCD7-2*. Asterisks indicate significant difference between both alleles: ***, **, *, and n.s. indicates *p* < 0.001, < 0.01, < 0.05, and ≥ 0.05, respectively (t-test)

We identified multiple QTLs for CD5 and CD4 in different regions from *qDTH3* (Fig. 3a–d). Among them, *qCD7-2* for CD5 and CD4 was identified in 24.57–26.64 Mb of the long arm of chromosome 7 in 2020 (Table 1). The same QTL for CD5 was also identified in 2021 (Table 1). Furthermore, *qSBN7* and *qSN7* were also located in the same position as *qCD7-2* (Table 1). The CD5 and CD4 in RILs having the Omachi allele of *qCD7-2* (RILs-*qCD7-2*^*OM*^) were significantly larger than in RILs with the Koshihikari allele (RILs-*qCD7-2*^*KO*^) in both years (Fig. 3e, f). The SBN and SN values in RILs-*qCD7-2*^*OM*^ were also significantly higher than in RILs-*qCD7-2*^*KO*^ (Fig. 3 g, h). In 2020, the total spikelet number per plant in RILs-*qCD7-2*^*OM*^ was higher than in RILs-*qCD7-2*^*KO*^ (Fig. 3j). In 2021, the total spikelet number per plant was similar between the alleles, because the enhanced SN in RILs-*qCD7-2*^*OM*^ was offset by the decreased PN (Fig. 3i and j).

Two other QTLs for CD5 and CD4were detected on the long arm of chromosome 3 (*qCD3*) and the short arm of chromosome 7 (*qCD7-1*) (Table 1). These two QTL were suggestive because the peaks of logarithm of the odds (LOD) values were detected in all analysis for CDs although they did not exceed the threshold in 2020. We also added *qCD1*, which located on chromosome 1, to calculate the effect of QTLs because the region showed higher LOD values than the surrounding regions for two consecutive years (Fig. 3a–d). Using the modeling approach, the total phenotypic variation explained (PVE) by the four QTLs for CD5 were 40.3% (2020) and 48.9% (2021), whereas the PVE by only significant QTL, *qCD7-2*, were 18.5% (2020) and 15.0% (2021) for CD5 (Additional file 2: Table S3). This indicates that these suggestive QTLs are also necessary to explain culm thickness of Omachi. Only *qCD1* had a negative additive effect, indicating that the Koshihikari allele enhances the culm thickness. The Omachi alleles increased the culm thickness in the other three QTLs.

**Fig. 4:**
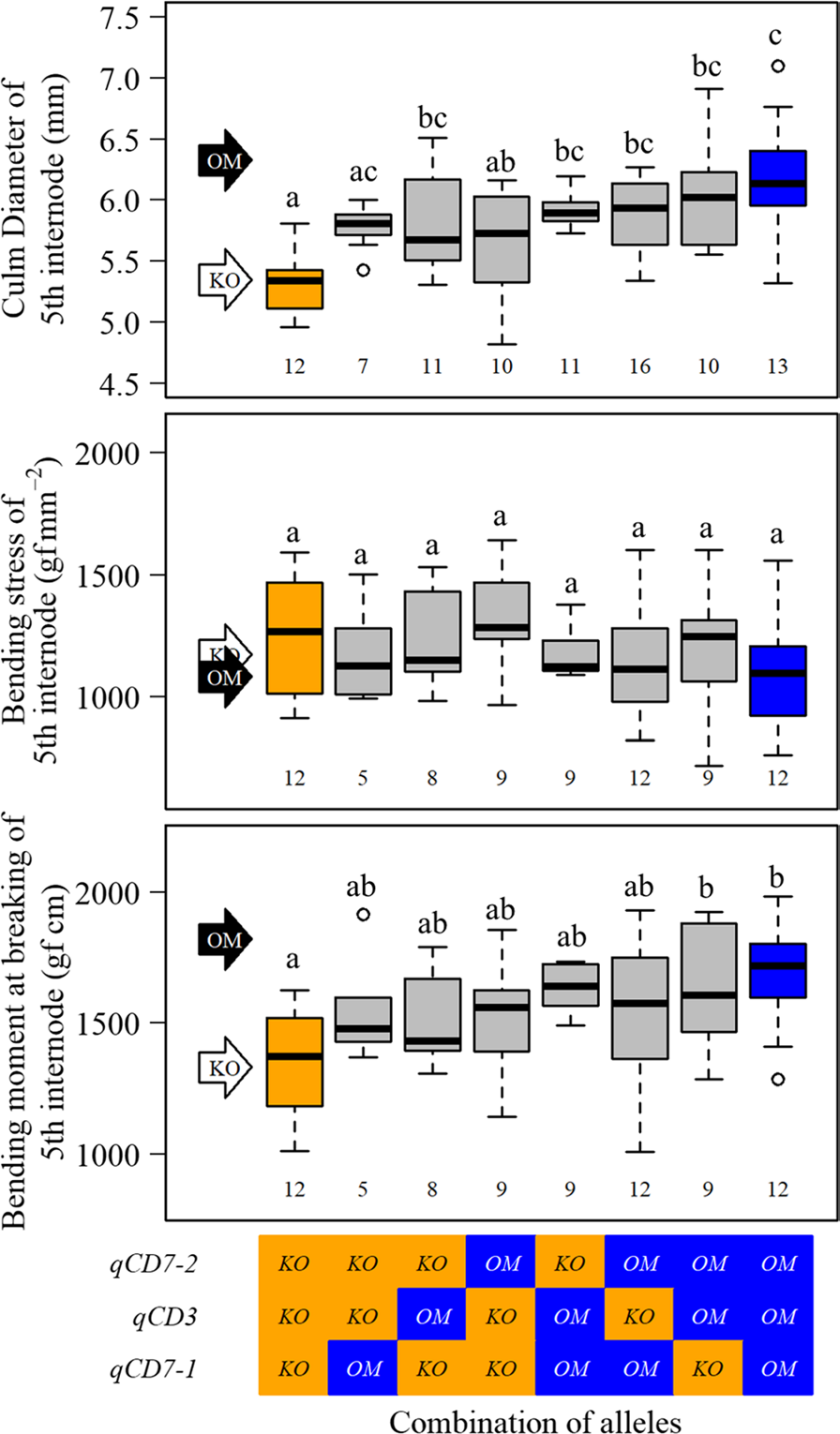
Comparison of the culm traits of the RILs classified on the basis of allele combinations. White and black arrows indicate the trait values of Koshihikari and Omachi, respectively. In boxplots, different letters indicate significant differences at *p* < 0.05 (Tukey’s test). The number under each boxplot indicates the number of RILs classified into the group

RILs were classified into eight groups on the basis of allele combinations in *qCD3*, *qCD7-1* and *qCD7-2*. In 2020, the mean values of CD5 were higher in groups with more Omachi alleles for these QTLs (Fig. 4). The RILs that accumulated the three Omachi alleles showed higher values of CD5 than the RILs with Koshihikari allele by + 0.89 mm (16.7%) (Fig. 4, Additional file 2: Table S5). These differences were almost equal to the differences in CD5 between Omachi and Koshihikari (0.98 mm) (Fig. 4, Additional file 2: Table S1). The enhanced CD5 led to an increase of 48.7% in SM of the 5th internode (Additional file 2: Table S5). BS tended to decrease slightly from the accumulation of the Omachi alleles, but there was no significant difference between groups (Fig. 4). BM was significantly higher (25.0%) in the RILs that accumulated the three Omachi alleles than in those with the Koshihikari alleles (Fig. 4, Additional file 2: Table S5). These results were almost same in 2021 (Additional file 1: Fig. S6).

The QTLs of TGW, GL and GW were not overlapped to any QTLs for CD (Table 1). This indicates that the culm thickness of Omachi is controlled by different genetic factors from grain size.

### QTL Analysis Using F_2_ Population Supporting Presence of *qCD7-2*

**Fig. 5:**
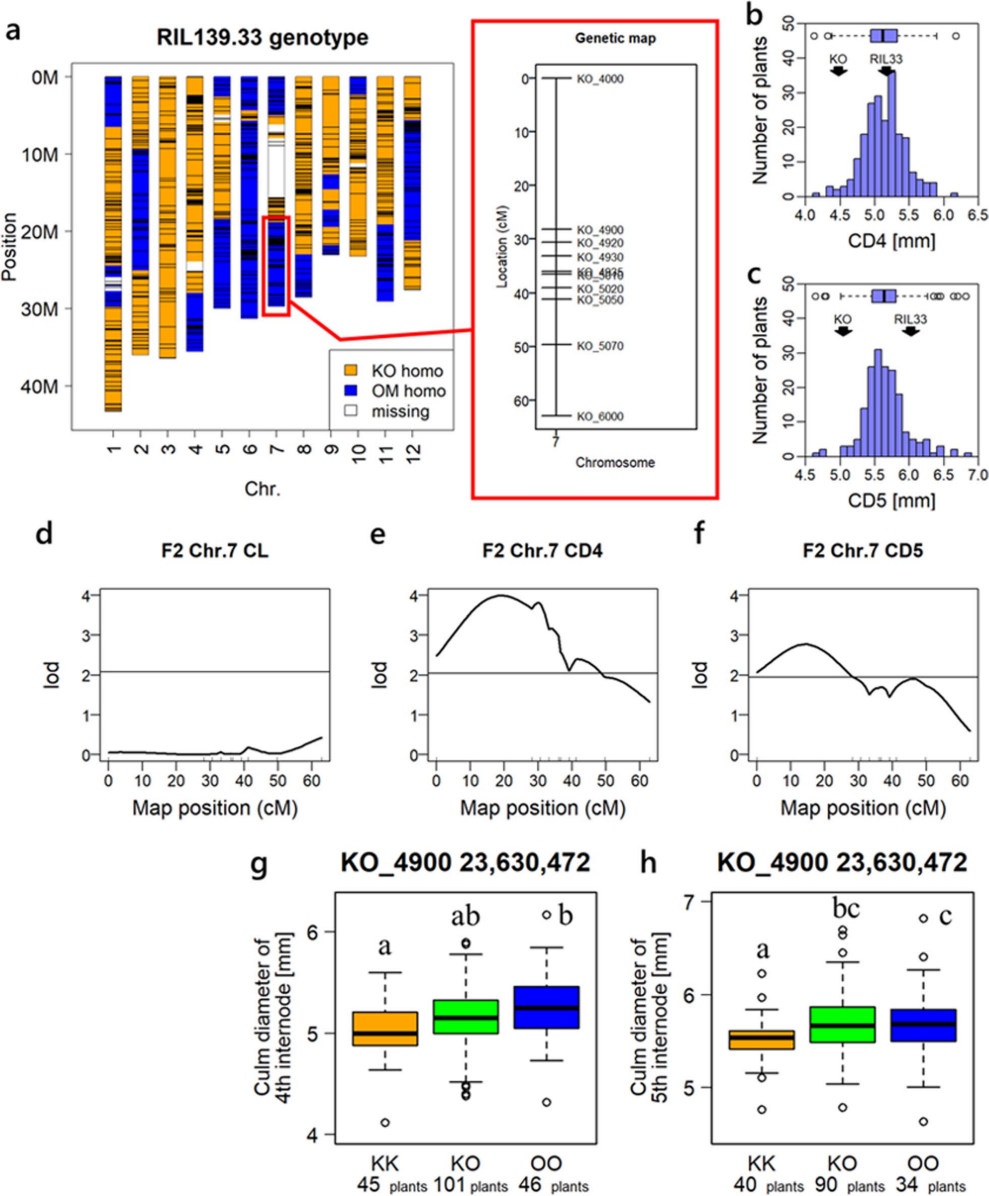
Results of QTL analysis in RIL33 × Koshihikari F_2_. **a** Genotypes of RIL33 genome and newly created DNA markers. **b**, **c** Histograms of culm diameter (**b** 4th internode, **c** 5th internode). **d**, **e** Results of QTL analysis in the long arm of chromosome 7 (**d** culm length, **e** culm diameter of 4th internode, **f** culm diameter of 5th internode). **g**, **h** Trait values of each group which were divided by genotype at the most related marker (KO_4900) to culm diameter. In boxplots, different letters indicate significant differences at *p* < 0.05 (Tukey’s test). 48 and 76 individuals in 240 individuals were omitted because their genotype or phenotype data were missing in the 4th and 5th internode, respectively. KK, KO and OO indicate homozygous of Koshihikari allele, heterozygous, and homozygous of Omachi allele, respectively

QTL analysis using the F_2_ population was conducted to verify the effects of *qCD7-2*. RIL line number 33, in which the region of *qCD7-2* was homozygous of Omachi allele, was crossed with Koshihikari and the F_1_ plants were self-propagated (Fig. 5a). We also created new PCR-based DNA markers to determine the genotypes of the F_2_ population on the long arm of chromosome 7 (Fig. 5b, Additional file 2: Table S5). The data of CD4 and CD5 among 240 individuals of F_2_ generation showed normal distributions (Fig. 5b, c). A QTL for both CD4 and CD5 was identified in the vicinity of marker KO_4900 on chromosome 7, while no QTL for CL was detected (Fig. 5d–f). The Omachi allele of *qCD7-2* increased the values of CD4 and CD5 relative to the Koshihikari allele (Fig. 5g, h).

### Candidate Causal Genes for QTLs

We examined the non-synonymous substitutions in the coding regions between Koshihikari and Omachi. 1,995 genes with mutations that cause changes in translation products in the whole genome were detected using the SNPEff. Of these, 68 genes and 2 genes were detected in the *qCD7-2* and *qCD3* region, respectively (Additional file 2: Table S6). Of these, 12 genes were classified as “HIGH” (causing frameshifts, STOP codons, etc.) with the greatest impact on translation products (Additional file 2: Table S7).

We also conducted RNA-seq analysis between Koshihikari and Omachi to compare differences in gene expression levels at the shoot apex during the vegetative stage and early reproductive stage (Additional file 2: Table S8). There were 224 (in 2020) and 350 (in 2021) differentially expressed genes (DEGs) during vegetative growth, and 770 (in 2020) and 1538 (in 2021) DEGs during early reproductive growth (Additional file 1: Fig. S7, Additional file 2: Table S8). In the genetic region of *qCD7-2*, there were 10 genes that were detected in more than two samples. Among them, four genes consistently showed different gene expression level in all years and growth periods (Additional file 2: Table S9). Of these four genes, we focused on three genes that may be responsible for CD.

**Fig. 6:**
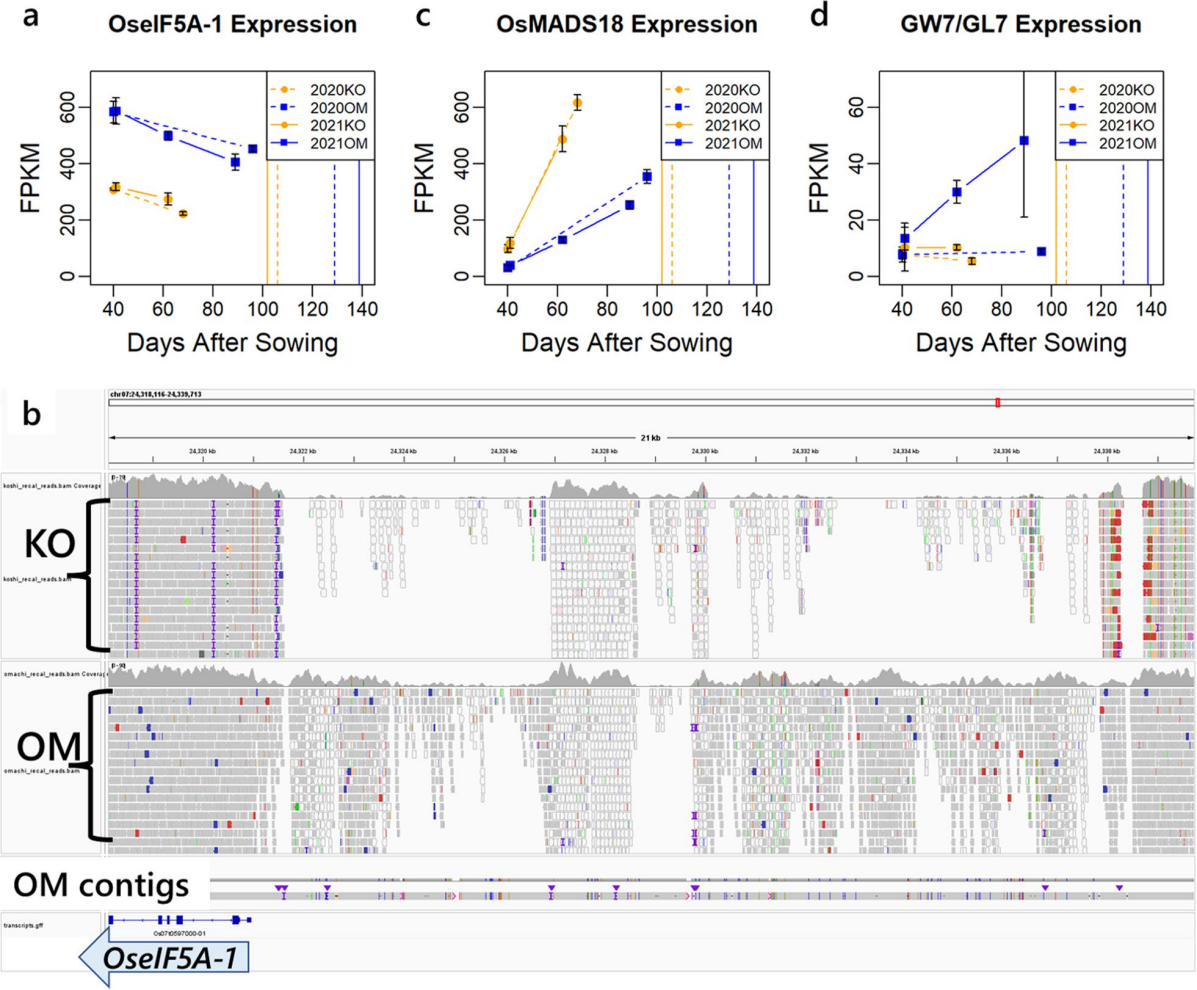
DEGs within the *qCD7-2* region. **a** Expression levels of *OseIF-5A*. Orange and blue lines indicate the expression levels in Koshihikari and Omachi, respectively. Dashed and solid lines indicate the expression levels in 2020 and 2021, respectively. **b** Mapping status of next generation sequence reads upstream of the *OseIF5A-1* gene coding region, visualized by Integrative Genomics Viewer (IGV). Top row: Koshihikari short reads, middle row: Omachi short reads, bottom row: contig composed of Nanopore long reads of Omachi. **c** Expression levels of *OsMADS18*. **d** Expression levels of *GW7*/*GL7*

The expression of *OseIF-5 A-1* (*Os07g0597000*) was approximately 1.4 to 2.0 times higher in Omachi over the two years at both the vegetative and early reproductive growth stages (Fig. 6a). The short read mapping of sequence data showed that there was a region where reads were not mapped normally in the upstream of *OseIF5A-1* (Fig. 6b). Furthermore, the contigs obtained by MinION long-read sequencing at Omachi mapped to the upstream region of *OseIF5A-1* differed greatly from the reference genome compared to other regions and were discontinuous (Fig. 6b). This indicated that there was a large structural variation in the upstream of *OseIF5A-1*.

The expression level of *OsMADS18* (*Os07g0605200*) was significantly higher in Koshihikari (Fig. 6c). *OsMADS18* is reported to be highly expressed in the shoot apical meristem and involved in tillering and heading (Yin et al. 2019). The expression level of *GW7*/*GL7*/*SLG7* (*Os07g0603300*) was significantly higher in Omachi in early reproductive growth (Fig. 6d). *GW7*/*GL7*/*SLG7* is reported to be a gene controls grain shapes (Wang et al. 2015a, b; Zhou et al. 2015).

## Discussion

### QTLs Responsible for Culm Thickness of Omachi

The distribution of CD in RILs showed a normal distribution, suggesting the existence of multiple QTLs (Additional file 1: Fig. S2). We identified three QTLs that increase culm thickness when they are Omachi alleles. Among these QTLs, *qCD7-2* has the greatest effect on CD and is the most reliable QTL because the LOD value of the region of *qCD7-2* exceeded the threshold in three of four QTL analyses for CD (CD4 in 2020 and CD5 in 2020 and 2021) (Fig. 3). In addition, the existence of *qCD7-2* was also verified by the QTL analysis using the F_2_ population derived from one of the RILs and Koshihikari. The other suggestive QTLs, *qCD3* and *qCD7-1* are also important factors in explaining culm strength of Omachi. However, further studies are needed to determine the exact regions and their effects on *qCD3* and *qCD7-1*. To verify the QTLs with relatively smaller effects than *qCD7-2*, it may be necessary to develop CSSLs and evaluate traits using populations with a uniform genetic background.

Whether accumulation of these QTLs improving CD actually leads to increased culm strength is of great interest. Our results showed that the RILs with Omachi alleles in *qCD3*, *qCD7-1* and *qCD7-2* had not only thicker culm, but also higher BM than in those with the Koshihikari alleles (Fig. 4). However, there is concern that this improvement of BM may be linked to *qDTH3* genotype because BM and BS were positively correlated to DTH, and the position of *qCD3* was close to *qDTH3* (Fig. 2a; Table 1). In actually, 19 RILs of the 23 RILs which had Omachi alleles in both *qCD7-2* and *qCD3* had Omachi allele in *qDTH3* (data not shown). However, considering that BM is the product of BS and SM, which calculated from CD, and there is no significant difference in BS among the set of genotypes (Fig. 4), accumulation of QTLs for CD is likely a factor of the increase in BM. In the future, this point must be verified by genetic analysis using populations with uniform DTH.

### Comparison With QTLs from Previous Studies

The heading date of Omachi was 23 days (2020) and 37 days (2021) later than that of Koshihikari, and the causal QTLs were detected as *qDTH3* and *qDTH6* (Table 1, Additional file 2: S1). These two QTLs contain the previously reported genes *Hd6*, *Hd16* and *Hd17* (Takahashi et al. 2001; Matsubara et al. 2012; Hori et al. 2013). The additive effects were largely consistent with that of previous studies, suggesting that the heading date of Omachi can be controlled by replacing alleles of these three genes. The alleles of Omachi in these three genes are the same as those of Yamadanishiki, the most widely used variety for *sake* brewing (Okada et al. 2017, 2018). For traits related to grains, QTLs such as *qTGW10*, *qTGW11*, *qGL11*, *qGW5*, and *qGW6* overlapped with the QTLs previously identified from Yamadanishiki (Okada et al. 2017, 2018). Yamadanishiki is derived from a cross between ‘Yamadaho’ and ‘Tankan-Wataribune’, which is presumed to have originated from Omachi (Yoshida 2012). Many of the QTLs detected in the population derived from a cross between Omachi and Koshihikari in this study coincided with QTLs detected in that of Yamadanishiki and Koshihikari. Thus, the alleles contributing to the characteristics of Yamadanishiki that make it suitable for *sake* brewing are likely to originate from Omachi.

While the QTLs for DTH, TGW, GL and GW were identical to those previously reported, the QTLs for CD have not been reported and are likely to be novel. In previous studies using GWAS of temperate *japonica* rice, QTLs for CD were reported on chromosome 5 (Chigira et al. 2020) and chromosomes 2, 6, 8, and 10 (Nomura et al. 2021). Although the populations used in both studies included Omachi, *qCD7-2* or other suggestive QTLs detected in this study have not been detected by GWAS.

### Candidate Genes for *qCD7-2*

*qCD7-2* spans about 4.7 Mb, which contains hundreds of possible causal genes. We used two methods to extract highly possible causal genes rapidly. First, 68 genes were extracted from SNPs and InDel information obtained from the next-generation sequencing data of Koshihikari and Omachi (Additional file 2: Table S6). Second, 10 genes were extracted from information on DEGs between parental varieties obtained by RNA-seq (Additional file 2: Table S9).

Of these, *OseIF-5 A-1* (*Os07g0597000*) extracted from the DEG information was considered particularly promising for a candidate causal gene. This gene encodes a transcription initiation factor-like protein eIF5A. The eIF5A is a highly conserved protein in eukaryotes and is the only protein containing the unique amino acid hypusine (Mehta et al. 1994). There are five eIF5A family genes in rice, one of which is *OseIF5A-1*. The expression level has been reported to change in response to environmental stresses in rice, suggesting that it contributes to stress tolerance (Chou et al. 2004). There are no reports of culm morphogenesis in rice, but studies in *Arabidopsis* have reported that the overexpression of a homologue of *OseIF5A-1* increases biomass and yield (Ma et al. 2010). It has also been reported that loss-of-function mutants have reduced sensitivity to cytokinins (Ren et al. 2013). If eIL5A also has a similar function in rice, it could affect culm morphogenesis. In the future, it is necessary to determine whether *OseIF5A-1* is the causal gene of *qCD7-2* using mutants and to analyze its physiological functions.

For another DEG, *OsMADS18* (*Os07g0605200*) was detected. It is reported that *OsMADS18* is highly expressed in the shoot apical meristem and is associated with tiller number and heading date (Fornara et al. 2004). It has also been reported that the overexpression of *OsMADS18* enhances *SCM3*/*FC1*, a strong culm-related gene (Yin et al. 2019) However, in this experiment, *OsMADS18* expression was significantly higher in Koshihikari with thin culms, and there was no significant difference in *SCM3*/*FC1* expression between Koshihikari and Omachi, so the relationship between *OsMADS18* expression and culm thickness is still unclear. The high expression of *OsMADS18* in Koshihikari has been reported to be due to gene duplication (Qin et al. 2021).

Notably, *GW7*/*GL7*/*SLG7* (*Os07g0603300*), a gene controlling rice grain shape, was detected as a DEG in *qCD7-2*. However, there was a large scattering in the expression levels among years and repetition. Further investigation is needed to obtain reliable data.

Although we have investigated several candidate genes mentioned above, there are still numerous candidate genes, and others may be the causal genes. Therefore, it is also necessary to simultaneously develop a population such that the QTL region can be narrowed down by the map-based cloning method.

### Pleiotropic Effects of QTLs

The QTLs of CD had pleiotropic effects on yield-related traits. Previous studies have revealed that the *SCM2*/*APO1* allele, which increases culm thickness, also increases the number of grains per panicle. This allele maintains the number of panicles and contributes to higher yields (Ookawa et al. 2010). In contrast, the *SCM3*/*FC1* allele similarly thickens culms and increases the number of grains per panicle, but decreases the number of panicles and has no effect on yield (Yano et al. 2015b). In the RILs used in this study, there was a significant positive correlation between CD and SN, although the correlation between CD and PN remained a weak negative (Fig. 2a). The Omachi allele of *qCD7-2* was found to be effective in increasing SN, but the extent to which PN decreased differed depending on the year (Fig. 3j). Therefore, it is not possible to determine from this result whether or not the Omachi allele of *qCD7-2* has a yield-enhancing effect. The effect on yield may also depend on other chromosomal regions genotypically different in each RIL. In the future, it will be necessary to verify this by developing a near-isogenic line in which only the *qCD7-2* region of Koshihikari is replaced with the Omachi allele.

### Usefulness of Landraces in Rice Genome Breeding Against Climate Change

While there have been studies that comprehensively analyzed the lodging resistance of temperate *japonica* rice landraces, few studies have compared individual landraces with breeding varieties (Chigira et al. 2020; Nomura et al. 2021). In this study, we conducted a one-to-one comparison of the phenotypes and genotypes between the strong culm landrace Omachi and the popular variety Koshihikari. We identified QTLs associated with culm thickness that were thought to be unique to Omachi for the first time.

Koshihikari, which was compared to Omachi in this study, was bred in 1956, and has been the most popular variety in Japan since 1979 (Kobayashi et al. 2018). Due to its immense popularity, Koshihikari and its progeny have often been used as crossing parents, resulting in a decline in genetic diversity (Yamamoto et al. 2010; Yonemaru et al. 2012). Moreover, Koshihikari has poor lodging resistance due to its thin and long culms (Ishimaru et al. 2008). For culm thickness in particular, few useful alleles have been found in temperate *japonica* subspecies, and little improvement has been made except for cases of gene transfer from *indica* and tropical *japonica* rice. In this study, we focused on Omachi, a landrace that has not been used to improve staple rice varieties, and clarified the position of QTLs affecting culm thickness. This suggested a path to improve the culm strength of modern temperate *japonica* cultivars by interbreeding within the subspecies.

The culm of Omachi is thick and strong, but the culm is long and prone to lodging. Moreover, the heading date of Omachi is later than those of varieties commonly grown today. Therefore, useful QTLs for culm strength should be independent of CL and DTH. In the RILs used in this study, CL and CD were independent, indicating that only culm thickness could be improved (Fig. 2a). In addition, although CL and other traits were highly correlated with DTH, CD had almost no correlation with DTH (Fig. 2b). QTLs detected for CD did not overlap with other traits (Table 1). Therefore, it should be possible to introduce the alleles enhancing culm thickness from Omachi without affecting CL or DTH. The QTLs identified in this study can be applied to strengthen the short culm varieties, which are widely grown in Asia.

There is a concern of trade-off that improvement in culm thickness may result in a decrease in PN. Certainly, there was a trade-off between CD and PN in our study, and some of the QTLs associated with CD were also detected in SN (Table 1). However, as mentioned above, it was suggested that genetic factors associated with CD increase SN rather than reduce PN pleiotropically in our analysis. Previous studies have shown that it is possible to overcome the trade-off between CD and PN and to achieve lodging resistance and increased yield by accumulating alleles that induce moderate morphological changes Yano et al. 2015b; Ookawa et al. 2022). Since the QTL related to culm thickness identified in this study also brings moderate changes in traits, accumulation with the previously identified superior alleles may contribute to improving lodging resistance without affecting yield or rather with increasing yield.

For these reasons, our identified QTLs may be useful for breeding rice varieties that are more resilient to climate change induced disasters by improving the lodging resistance of many varieties currently grown.

## Conclusion

In this study, QTLs responsible for culm thickness of temperate *japonica* varieties were identified using RILs derived from cross between Omachi and Koshihikari. Among of them, *qCD7-2* on the long arm region of chromosome 7 was stably detected and had the largest effect to the culm thickness. Japanese landrace rice Omachi had alleles strengthening culms without affecting culm length or heading date, while Omachi has been rarely used for the breeding of staple rice varieties. These results support the usefulness of the Omachi alleles of these QTLs for improving lodging resistance of modern varieties through genomic breeding with marker assisted selection. In the future, further research is expected to identify the genes responsible for these QTLs, and it will lead to a better understanding in the mechanism of culm strength.

## Materials and methods

### Plant Material and Cultivation

The rice (*Oryza sativa* L.) variety, Koshihikari and Omachi were cultivated in 2020 and 2021 to compare their traits. Field experiments were conducted in a paddy field in the Field Museum Honmachi, Field Science Center, Faculty of Agriculture, Tokyo University of Agriculture and Technology. The seeds were sown in paper pots (Nippon Beet Sugar Manufacturing Co., Ltd. Hokkaido, Japan) on April 22, 2020, and April 21, 2021. The seedlings at the four-leaf stage were transplanted to a paddy field on May 14, 2020, and May 13, 2021, with one seedling per hill. The planting density was 22.2 hills m^−2^, and the spacing was 15 × 30 cm. Each plot had 50 hills (1.5 × 1.5 m). For fertilizer, N, P_2_O_5_ and K_2_O were applied at 50 kg ha^−1^, 60 kg ha^−1^ and 60 kg ha^−1^, respectively. Weed and pest control were performed as needed. The fields were always under irrigated conditions. The number of replications was set to 3 for 2020 and 6 for 2021, and each plot was evenly distributed throughout the field.

For QTL analysis, RILs derived from Koshihikari × Omachi crosses (F_14_ in 2020, F_15_ in 2021, *n* = 96) were cultivated in 2020 and 2021. Cultivation conditions were as described above, with one replication of each line. In 2021, the array of each line in the field was shuffled. In 2021, the F_2_ population consisting of 240 individuals was also cultivated. This population was derived from crossing between one of the RILs (line number ‘RIL33’, its genotype is shown in Fig. 5a) and Koshihikari in the summer of 2020. The F_1_ generation was grown in the winter of 2020 to 2021, to get F_2_ seeds using a green house.

### Phenotyping

The heading date of the main culms for each variety were recorded and they were sampled 15 days after heading. Before sampling, the number of panicles of eight consecutive plants was counted, and the average of the counts was used as PN. For the parent varieties and RILs, 6–8 main culms that had an average length of the basal internode were chosen and used for phenotyping. Morphological traits, including CL, PL, AL, FL, primary branch number, SBN and SN were measured for each sample. For the physical parameters associated with culm strength, we recorded BM of 4th and 5th internodes using a Tensilon RTG-1210 universal testing machine (A&D, Tokyo, Japan). We also measured the culm thickness of 4th and 5th internodes by assuming the section to be an ellipse with a hollow shape using an image analysis software ‘Smeasure’ (https://github.com/KChigira/Smeasure), which was written using OpenCV v3.4.5 (https://github.com/opencv/opencv). The outer diameter of the major axis was used as CD. From these parameters, BS were calculated using the following formula, based on the method of Ookawa and Ishihara (1992).

1 $$M=\sigma Z$$

2 $$Z=\frac{\pi \left({{a}_{1}}^{3}{b}_{1}-{{a}_{2}}^{3}{b}_{2}\right)}{32{a}_{1}}$$

(1) $M$ is the bending moment at breaking (BM), $\sigma$ is the bending stress (BS), and $Z$ is the section modulus (SM). (2) ${a}_{1}$ is the outer diameter of the minor axis, ${b}_{1}$ is the outer diameter of the major axis (CD), ${a}_{2}$ is the inner diameter of the minor axis, and ${b}_{2}$ is the inner diameter of the major axis.

The plants were harvested at over 40 days after their heading date, and the harvested grains were air-dried and dehulled. About 10 g grains were sampled excluding broken and immature grains, and traits including number of grains, GL, GW and white core rate were measured using a grain discriminator ES-5 (Shizuoka Seiki Co., Ltd. Shizuoka, Japan) In the ES5 machine, white core and milky white grains are separately classified. The weight of the grains was measured and divided by the number of grains to obtain the TGW.

For the F_2_ population, only CL and CDs were measured using the methods mentioned above.

### Genotyping

Genotype of the RILs were analyzed using a GoldenGate® Assay (Illumina, CA, USA) according to the manufacturer’s instructions. We also conducted genotyping using GRAS-Di® analysis (TOYOTA, Aichi, Japan). Combining these two data, we got genotypes on 1,904 SNP markers (Additional file 1: Fig. S4, Additional file 2: Table S10). Genotype of the F_2_ population were analyzed by PCR-based DNA markers (n = 10) which is newly made based on next generation sequencing data of Koshihikari and Omachi (Additional file 2: Table S5).

### QTL Analysis Using RILs and F_2_ Population

For the RILs, linkage maps were constructed using the ‘est.map’ function of R/qtl software (Broman et al. 2003). QTL analysis was also conducted with R/qtl software, using the composite interval mapping method. The critical threshold values of LOD score were calculated by conducting 1,000 permutation tests with a significance level at *P* = 0.05. For the F_2_ population, we used the simple interval mapping method. Other conditions are same as RILs.

### Detection of Candidate Variants

Sequence reads of Koshihikari and Omachi were obtained from the European Nucleotide Archive; ID: DRX002963, DRX000450, respectively. Low-quality bases and the adaptors in the sequence reads were trimmed with Trimmomatic software (Bolger et al. 2014). Trimmed reads were mapped to the Nipponbare reference sequence, IRGSP-1.0 using BWA software (Li and Durbin 2010). Mapped reads were sorted and PCR duplication marked using GATK software (https://github.com/broadinstitute/gatk/). To improve the raw alignment, base quality score re-calibration was performed using GATK software. SNPs and indels were identified individually for each sample using GATK software. From the SNPs and indels data, we extracted the DNA mutations that cause mutations in the coding region using SNPEff (Cingolani et al. 2012).

### RNA-seq

Shoot apex of Koshihikari and Omachi were sampled in the sampling date shown in Additional file 2: Table S8. There were three repetitions derived from different plot. Tissue from two or three individuals were bulked for each repetition. Sample tissues were collected between 9:00 a.m. and 12:00 a.m. and immediately frozen in liquid nitrogen. Samples were ground with zirconia beads, using the TissueLyser (QIAGEN, MD, USA) with the adapters chilled by liquid nitrogen. Total RNA was extracted by Maxwell 16 LEV Plant RNA Kit (Promega, WI, USA) according to the manufacturer’s instructions. The amount of RNA was determined using Qubit RNA BR Assay Kit (Thermo Fisher Scientific, Waltham, MA, USA). Library preparation was performed according to the Lasy-Seq method (Kamitani et al. 2019). The libraries were sequenced by PE 150 sequencing with Illumina platform. The expression levels of each gene were estimated by RSEM software (Li and Dewey 2011). Compare of expression levels of each genes between Koshihikari and Omachi, and detection of DEGs were performed using the edgeR software (Robinson et al. 2009).

### Statistical Analysis and Visualization

Two-tailed Welch’s t-test was performed using the ‘t.test’ function of R software (version 4.1.3). The correlation coefficients between phenotypic trait values were calculated using the ‘cor’ function of R software. The multiple comparison tests were performed using the ‘TukeyHSD’ function of R software. The next generation sequence data was visualized by Integrative Genomics Viewer (IGV) (Robinson et al. 2011).

## Supplementary Information

**Additional file 1**. **Figure S1.** Average of daily temperature and sunshine duration during the experiment (AMeDAS in Fuchu). **Figure S2.** Distribution of the values of each trait in Koshihikari × Omachi RILs. **Figure S3.** Correlation matrix among all traits in Koshihikari × Omachi RILs. **Figure S4.** Genetic map of 1904 SNP markers used for QTL analysis. **Figure S5.** Mutations in genes related to heading date between Omachi and Koshihikari. **Figure S6.** Comparison of traits associated with culm strength for the RILs classified according to combination of alleles at *qCD3*, *qCD7-1* and *qCD7-2* in 2021. **Figure S7.** Detection of differently expressed genes (DEGs) between Omachi and Koshihikari by RNA-seq in shoot apex.

**Additional file 2**. **Table S1.** Comparison of the traits between Koshihikari and Omachi. **Table S2. **Summary of trait values in Koshihikari × Omachi RILs. **Table S3.** The estimation of effects of QTLs using a model constructed by the genotypes of four QTLs for culm diameter. **Table S4.** Information on markers used for genotyping RIL33 × Koshihikari F_2_. **Table S5.** Average of the trait values in each group of RILs classified according to combination of alleles at *qCD3*, *qCD7-1* and *qCD7-2*. **Table S6.** The number of variants classified 'HIGH' or 'MODERATE' by the SNPEff. **Table S7.** Detailed information of genes which include mutations classified ‘HIGH’ by the SNPEff software. **Table S8.** Collection date of samples used for RNA-seq and number of DEGs detected for each sample. **Table S9.** Detail information about DEGs detected in *qCD7-2*. **Table S10.** Information of markers used in QTL analysis.

## Data Availability

The datasets supporting the conclusions of this article are included within the additional files.

## Abbreviations

QTL: Quantitative trait locus
CSSL: Chromosome segment substitution line
BM: Bending moment at breaking
SM: Section modulus
GWAS: Genome-wide association study
PVE: Phenotypic variance explained
CL: Culm length
PL: Panicle length
AL: Awn length
FL: Flag leaf length
SBN: Secondary branch number
SN: Spikelet number per panicle
CD: Culm diameter
CD5: Culm diameter of 5th internode
CD4: Culm diameter of 4th internode
TGW: Thousand grain weight
GW: Grain width
GL: Grain length
RIL: Recombinant inbred line
DTH: Days to heading
LOD: Logarithm of the odds
DEG: Differentially expressed gene
