# Supplementary material for: Identification of Novel Quantitative Trait Loci for Culm Thickness of Rice Derived from Strong-Culm Landrace in Japan, Omachi
Source: Rice (N Y). 2023 Jan 27;16:4. doi: 10.1186/s12284-023-00621-8 (PMC9883377; doi:10.1186/s12284-023-00621-8)
Supplement: Supplementary file 1 — Additional file 1. Figure S1. Average of daily temperature and sunshine duration during the experiment (AMeDAS in Fuchu). Figure S2. Distribution of the values of each trait in Koshihikari × Omachi RILs. Figure S3. Correlation matrix among all traits in Koshihikari × Omachi RILs. Figure S4. Genetic map of 1904 SNP markers used for QTL analysis. Figure S5. Mutations in genes related to heading date between Omachi and Koshihikari. Figure S6. Comparison of traits associated with culm strength for the RILs classified according to combination of alleles at qCD3, qCD7-1 and qCD7-2 in 2021. Figure S7. Detection of differently expressed genes (DEGs) between Omachi and Koshihikari by RNA-seq in shoot apex. [file 12284_2023_621_MOESM1_ESM.pptx]

## Slide 1
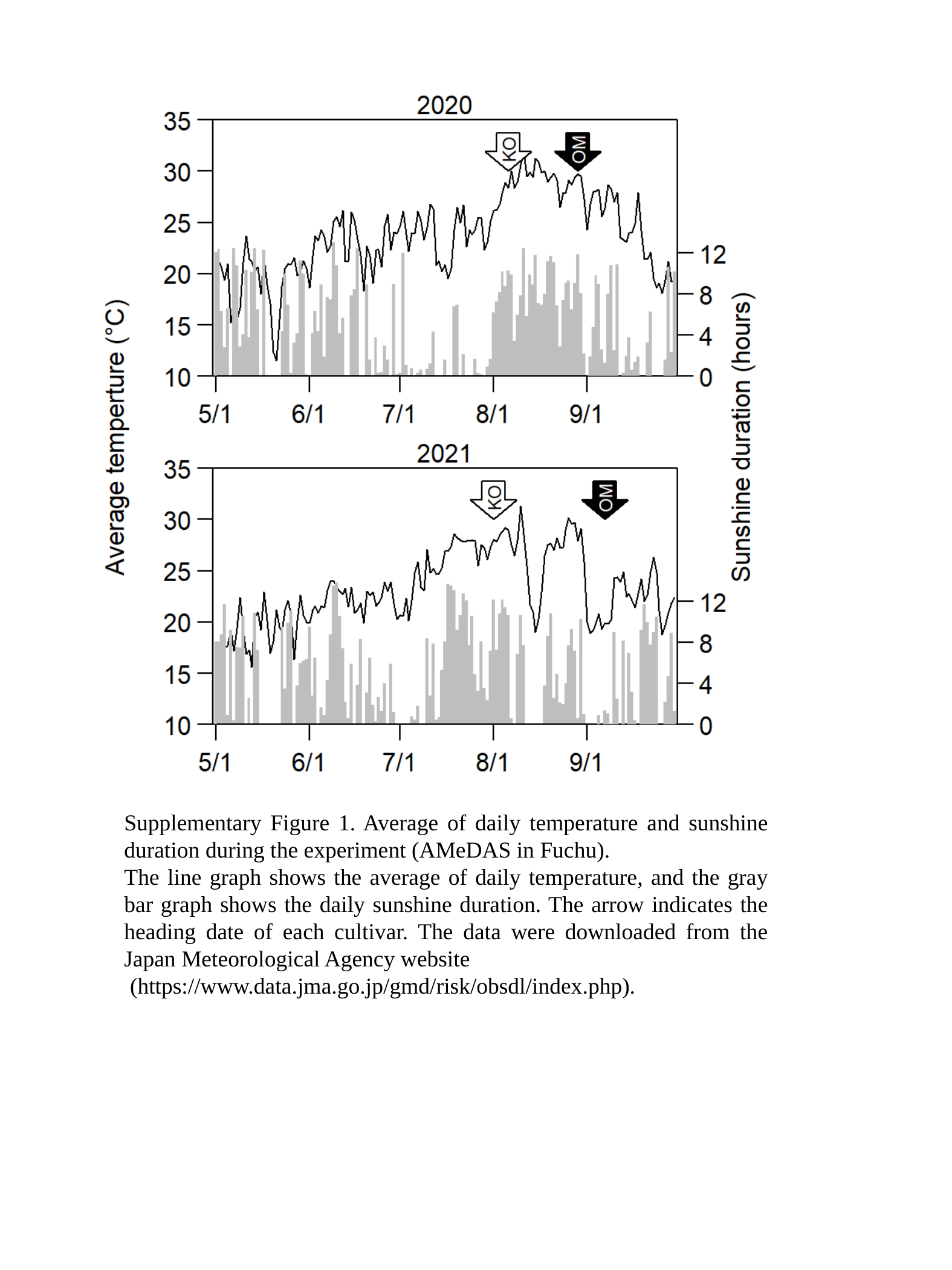

Supplementary Figure 1. Average of daily temperature and sunshine duration during the experiment (AMeDAS in Fuchu).
The line graph shows the average of daily temperature, and the gray bar graph shows the daily sunshine duration. The arrow indicates the heading date of each cultivar. The data were downloaded from the Japan Meteorological Agency website
 (https://www.data.jma.go.jp/gmd/risk/obsdl/index.php).

## Slide 2
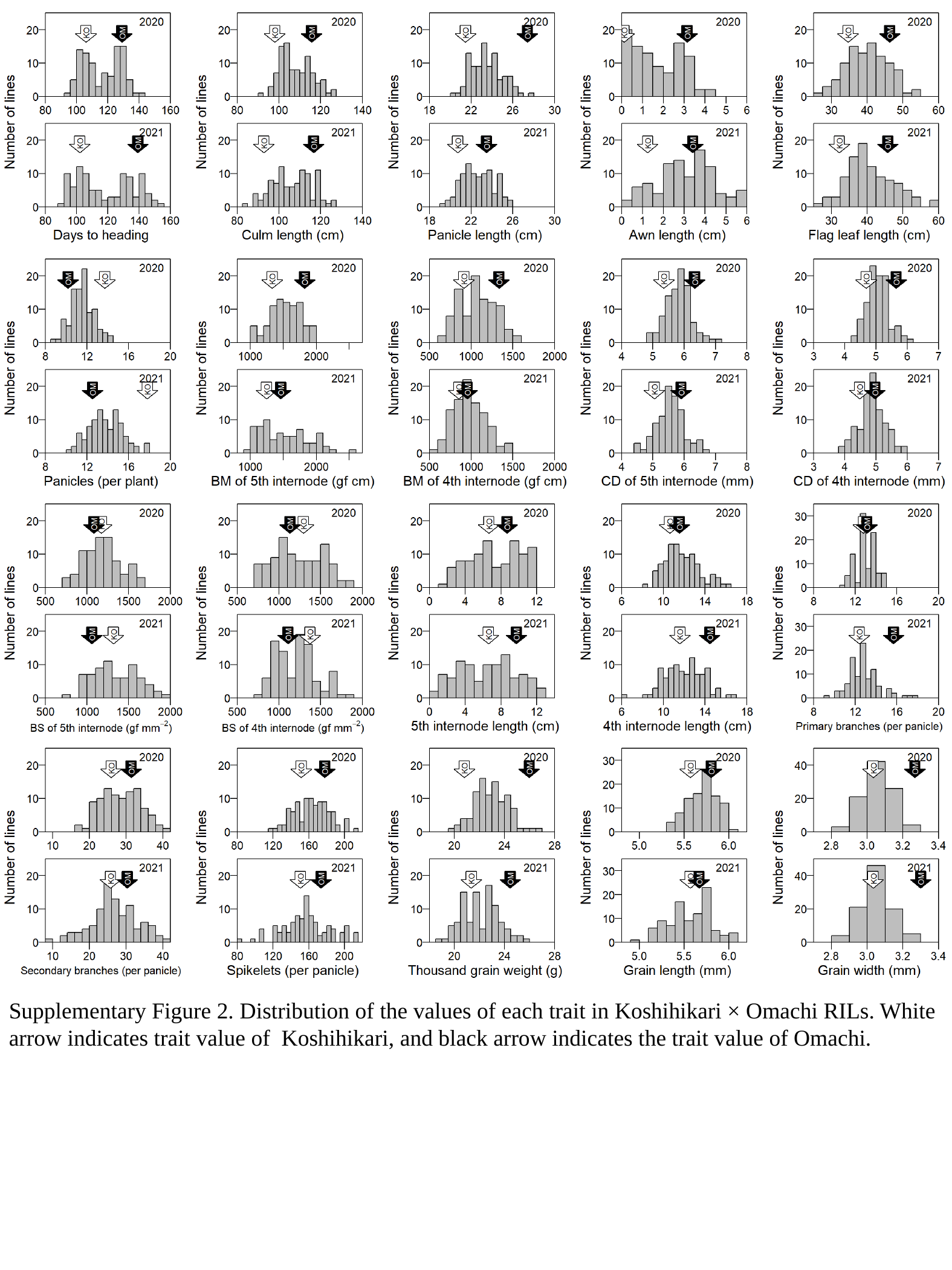

Supplementary Figure 2. Distribution of the values of each trait in Koshihikari × Omachi RILs. White arrow indicates trait value of Koshihikari, and black arrow indicates the trait value of Omachi.

## Slide 3
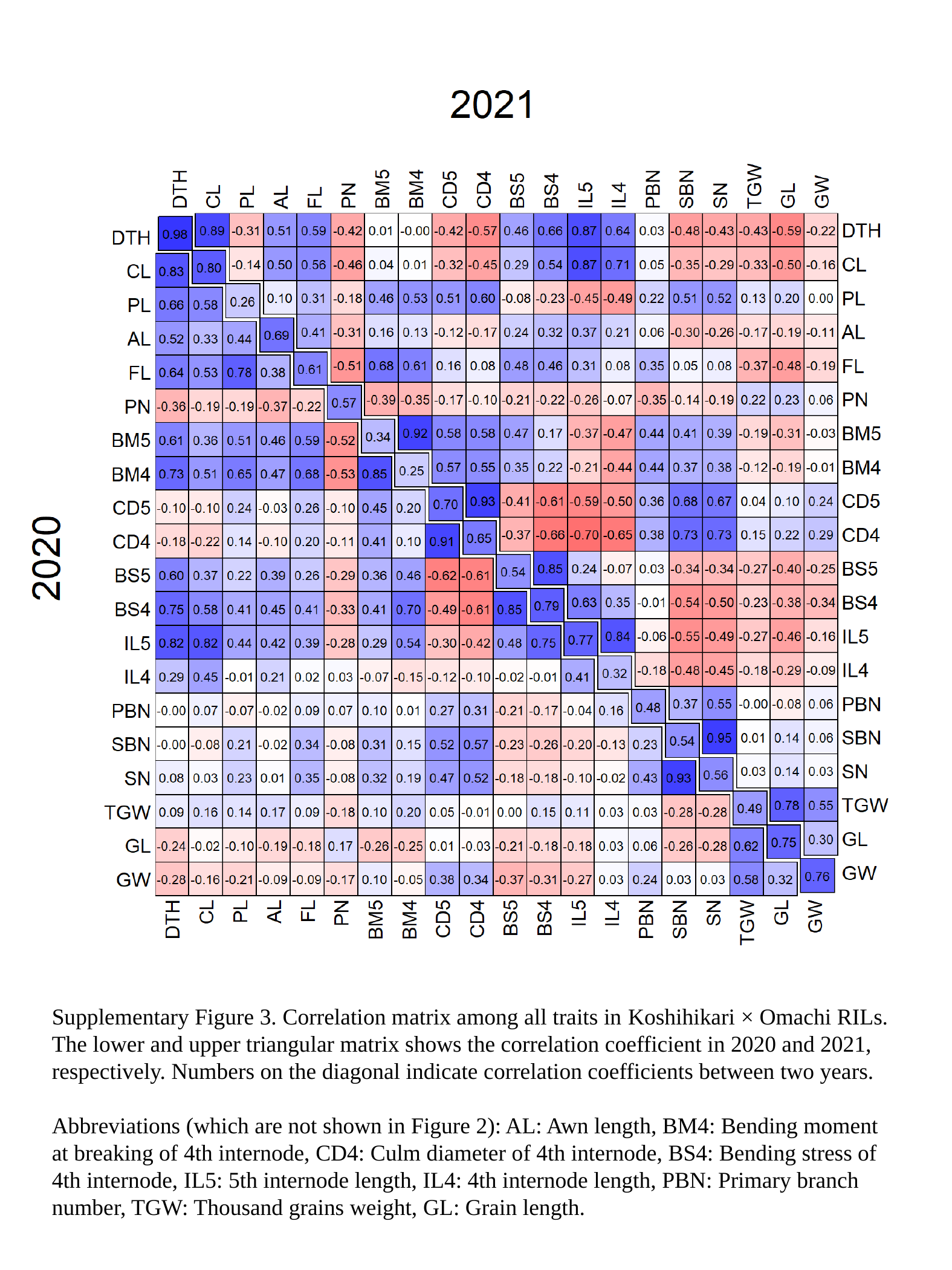

Supplementary Figure 3. Correlation matrix among all traits in Koshihikari × Omachi RILs. The lower and upper triangular matrix shows the correlation coefficient in 2020 and 2021, respectively. Numbers on the diagonal indicate correlation coefficients between two years.
Abbreviations (which are not shown in Figure 2): AL: Awn length, BM4: Bending moment at breaking of 4th internode, CD4: Culm diameter of 4th internode, BS4: Bending stress of 4th internode, IL5: 5th internode length, IL4: 4th internode length, PBN: Primary branch number, TGW: Thousand grains weight, GL: Grain length.

## Slide 4
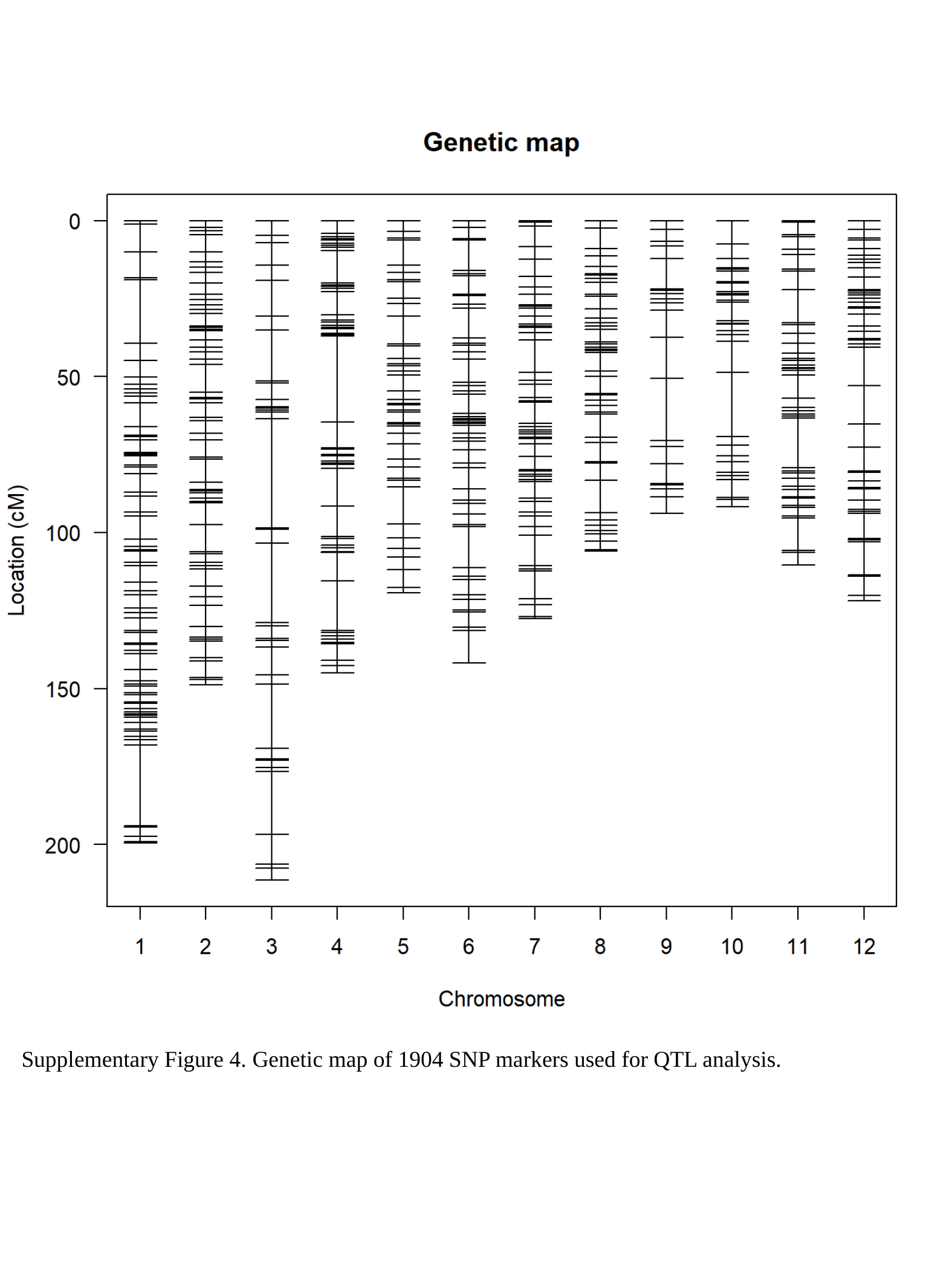

Supplementary Figure 4. Genetic map of 1904 SNP markers used for QTL analysis.

## Slide 5
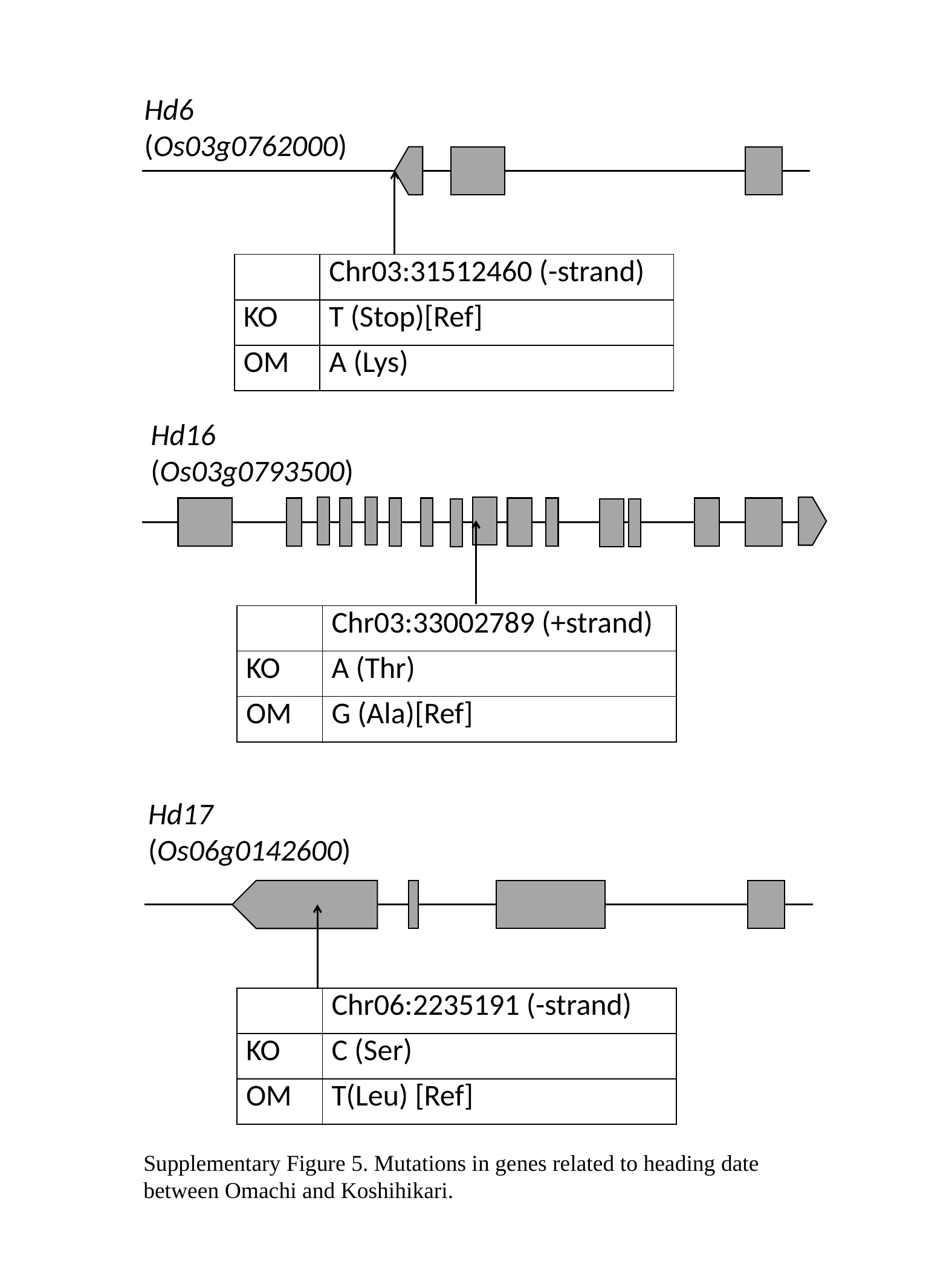

Hd6
(Os03g0762000)
| | Chr03:31512460 (-strand) |
| --- | --- |
| KO | T (Stop)[Ref] |
| OM | A (Lys) |
Hd16
(Os03g0793500)
| | Chr03:33002789 (+strand) |
| --- | --- |
| KO | A (Thr) |
| OM | G (Ala)[Ref] |
Hd17
(Os06g0142600)
| | Chr06:2235191 (-strand) |
| --- | --- |
| KO | C (Ser) |
| OM | T(Leu) [Ref] |
Supplementary Figure 5. Mutations in genes related to heading date between Omachi and Koshihikari.

## Slide 6
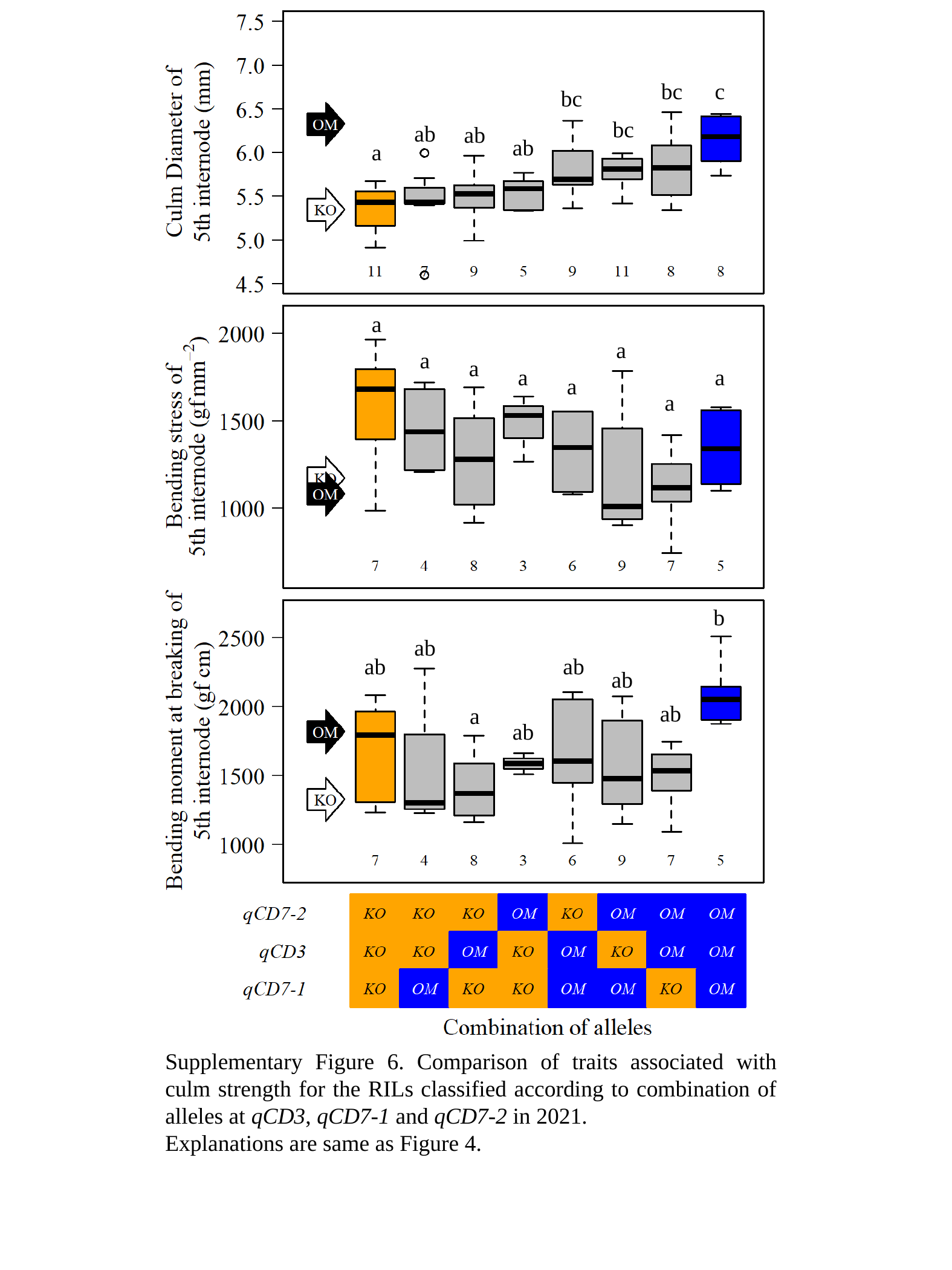

bc
c
bc
bc
ab
ab
ab
a
a
a
a
a
a
a
a
a
b
ab
ab
ab
ab
ab
a
ab
Supplementary Figure 6. Comparison of traits associated with culm strength for the RILs classified according to combination of alleles at qCD3, qCD7-1 and qCD7-2 in 2021.
Explanations are same as Figure 4.

## Slide 7
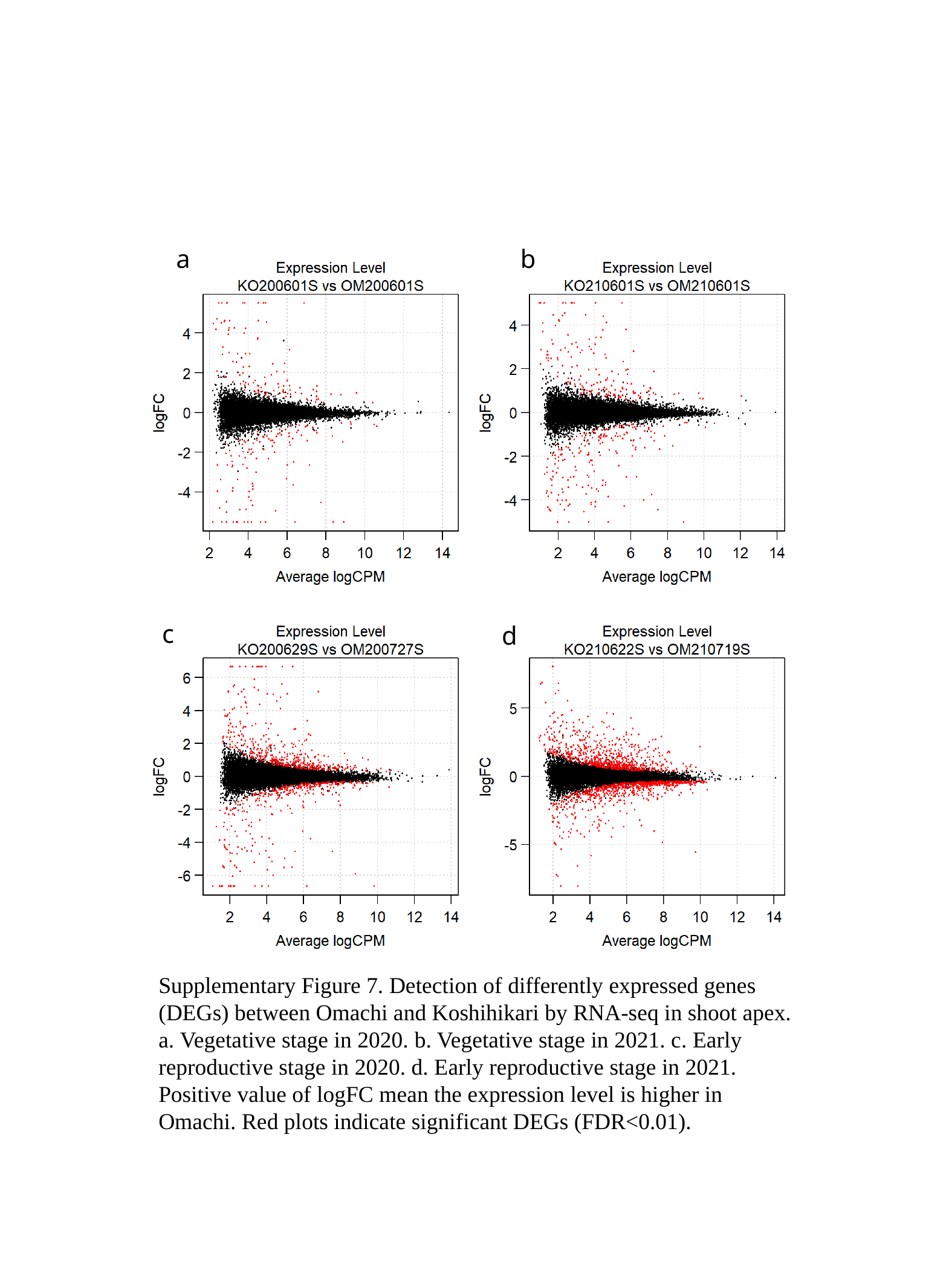

a
b
c
d
Supplementary Figure 7. Detection of differently expressed genes (DEGs) between Omachi and Koshihikari by RNA-seq in shoot apex. a. Vegetative stage in 2020. b. Vegetative stage in 2021. c. Early reproductive stage in 2020. d. Early reproductive stage in 2021. Positive value of logFC mean the expression level is higher in Omachi. Red plots indicate significant DEGs (FDR<0.01).
